# Reopening borders: protocols for resuming travel during the COVID-19 pandemic

**DOI:** 10.6061/clinics/2021/e2723

**Published:** 2021-03-01

**Authors:** Luiz Henrique da Silva Nali, Felipe Scassi Salvador, Graciela dos Santos Soares Bonani, Heitor Franco de Andrade Júnior, Expedito José de Albuquerque Luna, Dennis Minoru Fujita

**Affiliations:** I Programa de Pos-Graduacao em Ciencias da Saude, Universidade de Santo Amaro, Sao Paulo, SP, BR; II Grupo de Pesquisa Epidemiologica, Aspectos Moleculares e Celulares das Doencas Infecciosas - CNPQ/UNISA, Sao Paulo, SP, BR; III Centro Universitario das Americas (CAM), Sao Paulo, SP, BR; IV Laboratorio de Epidemiologia, Instituto de Medicina Tropical, Universidade de Sao Paulo, Sao Paulo, SP, BR; V Laboratorio de Investigacao Medica (LIM49), Faculdade de Medicina FMUSP, Universidade de Sao Paulo, Sao Paulo, SP, BR

According to the World Health Organization, COVID-19 has affected 28.04 million people resulting in 906,092 deaths in 216 countries as of September 11, 2020 (1). As a respiratory virus infection, transmission occurs by direct or indirect contact, usually by droplet spray in a short range** **or by aerosol in long-range transmission.

Various international health government agencies, such as the Center for Disease Control and Prevention and the World Health Organization, advice to maintain a safe distance of 6 feet between persons to avoid the respiratory droplets produced while talking, sneezing, or coughing. Droplets >5 μm, have a reduced life-time in air and travel a smaller distance (2).

The study of Setti et al. indicated that a distance of 6 feet is safe only if everybody wears face masks (3) to avoid the transmission of the virus in aerosolized droplets. Coronaviruses may be viable in air for up to 3 hours, and up to 72 hours on plastic or steel surfaces, and the virus can be carried by air-conditioned systems over long distances (4) or remain in areas with high contamination, such as hospitals. The virus was found to be widely distributed in the air and on surfaces in an area up to 3.94 meters or 13 feet, according to a study developed in hospital wards (5).

A few studies presented other possibilities, including the contamination of surfaces, such as remote controls, computer mouse, cardboard, exercise equipment, and even the floor of hospitals (5).

The transmissibility, high survival capacity in the environment, slow process of classification/diagnosis, and delay in the establishment of preventive/containment measures led to the initial rapid spread of the virus to other cities near Wuhan.

The delay in the surveillance and control along with the high number of international travels worldwide and mass gatherings (6) resulted in the infectious disease becoming a pandemic. The Diamond Princess cruise ship exemplified this concern with the rapid infection of passengers on-board during the quarantine (7).

## COVID-19 and tourism activity: protocols for domestic reopening

According to UNWTO data (8), the pandemic led to a reduction of approximately 57% in international arrivals in March and a consequent total halt in the following two months, with a reduction of 850 million to 1.1 billion international travels. It may represent a loss of $ 910 billion to US $ 1.2 trillion in export revenues in addition to the closure of direct tourism jobs with a dismissal of 100 to 120 million employees worldwide.

The preventive measures established in all the affected countries after the recognition of COVID-19 relied on old, but functional methods, such as quarantine, primary care practices, such as self-hygiene protocols composed of a constant practice of hand sanitizations, use of masks, being alert to the disease symptoms, and others. Social isolation in the absence of vaccines, clinical protocols for the care of critically ill patients, and the repositioning of previously known drugs were implemented.

These measures have been adopted by some nations in addition to modified travel practices with additional protocols, such as the screening of travelers’ body temperature and sanitation tunnels for possible external disinfection of individuals before entering airports, hotels, and event rooms; establishments operating with a reduced service capacity to increase the safety distance between customers in confined spaces, such as restaurants, concert halls, and other places of potential mass gathering conditions.

Some hotels established new hygiene protocols in the form of personal protective equipment for service teams with the use of disposable gloves, masks and face shields, increased cleaning cycles in common areas, sterilization of rooms by UV light or Oxy Sanitizer, high availability of 70% alcohol gel, free disposable masks for guests, use of more disinfectants, such as the use of chlorine-based solutions, removal of superfluous furniture and equipment from the rooms, among other methods that facilitate an easy cleaning process and reduce the risk of contamination of guests and workers.

However, there is no complete guarantee for safety despite taking these measures given the current scenario and knowledge about COVID-19; for example, the study of Olsen et al. (9), that presents the mobile phone, an equipment that most people have and carry everywhere, as a ‘Trojan horse' contributing to the transmission of microbial infections during epidemics and pandemics.

We also emphasize that some of the methods previously mentioned have been ineffective in past epidemics as well as during the current pandemic. These include temperature screening at airports (10), the disinfection of individuals by spraying in chambers or tunnels (11), and others.

The reopening of travel and entertainment services, despite the adoption of these preventive measures, should only occur in the absolute decline of COVID-19 confirmed cases and deaths, with the reinforcement of preventive practices to reassure the reduction of the disease incidence. In São Paulo State, for example, after the Brazilian Independence Holiday, there was an increase of 8.99% and 8.05% in COVID-19 incidence and deaths, respectively, in several cities due to domestic trips and parties (12).

A protocol of constant surveillance and a sentinel system, a tool well-known to health experts and wisely handled by them (13), should be established in local destinations and tourism services, to assist health authorities in controlling potential outbreaks and the spread to other regions.

## COVID-19 and International Travels: The major problem

Some countries support the idea of an “immunity passport” or a “risk-free certificate” to allow people who present antibodies against SARS-CoV-2 and could be protected against re-infection to travel. However, there is no evidence, until now, whether the antibodies induced by SARS-CoV2 infection could prevent further infection and for what duration (14).

However, through epidemiological data related to the incidence and mortality, countries can strengthen their border policies with the permission or refusal of travelers from highly endemic regions as well as recommending that their citizens travel only to destinations considered safe.

In this way, the countries with an elevated incidence plateau or with unclear data can be included in this high-risk classification, and travelers coming from them should be barred, as recommended in Article 18-Item 2 of the International Health Regulations (WHO, 2005).

As of September 11, 2020, 4,282,164 cases had been reported in Brazil, with 130,396 total deaths, with an incidence of 2,037.7 per 100,000 inhabitants (15) and a high R_t_ of 0.94 (16), representing a potential risk for international security**.**

The country will probably be avoided for a long time by international travelers due to the president’s negligent behavior that has resulted in the absence of a public health policy for pandemic management.

In Europe, this border situation is more complicated due to the size of the countries and their close and easily accessible frontiers (akin to domestic travel), thereby presenting a great risk in case of reopening travel activities, even in a controlled epidemic condition (17).

In some regions, the reemergence of COVID-19 is led by the summer vacation movement to countries on the Mediterranean coast, such as Spain. The quick intervention of local authorities, surveillance, and isolation of cases, with a responsible public health policy and strategy, may arrest the incidence and deaths by COVID-19.

## Recommendations for the return of domestic and international travels

In order to reopen international travel, governments need to revise their public health policies to enable enhanced surveillance, tracking and containment of confirmed cases, management of patients, treatment, and support infrastructure, among other actions necessary to reduce the incidence of cases and deaths.

The United States and Brazil are countries that currently lead in the number of confirmed cases worldwide. Both nations did not consider the scientific evidence from the initial studies, not adopting preventive and protective strategies, which could have considerably reduced the number of cases and deaths.

In Brazil, unfortunately, the federal government handled the COVID-19 issue carelessly and reinforced the false message that it is an ordinary and treatable disease.

Due to this situation, several countries will keep their borders closed to receive citizens of nations that have their epidemiological situation undefined for COVID-19, including Brazil, for the reasons mentioned previously. This situation may last for a long time, as there is still no effective public health policy against COVID-19 in the country.

The adoption of quarantine is a way to allow the entry of travelers from these regions; however, only those with high purchasing power will be able to comply with this regulation.

We emphasize that several travelers, despite being a part of these restricted nations, have dual citizenship, which allows them to travel freely to countries with restrictions for certain nationalities.

The establishment of mandatory quarantine with the surveillance of personal mobile phones and online social networks to verify social isolation, online medical and psychological counseling in the isolation period, fines for breaching travel quarantine rules, among other measures may reduce these risks for COVID-19 importation.

Although there are several candidates for COVID-19 treatment, none have been approved. There are no vaccines against SARS-CoV2, but in the future, a vaccine certificate may be required at the border controls.

The introduction of visas with a pre-approved itinerary in this time may be an important preventive measure for surveillance in case a traveler is positive for COVID-19, and may protect the destination regions and travel establishments from exposure.

Another important tool is the testing of travelers when they arrive at a destination. The results may allow the screening of travelers who arrived by the same transport, thereby avoiding the high transmission of COVID-19 that may occur at the border control.

Therefore, protective measures, such as social distance are mandatory to avoid further dissemination of the virus. In addition, other ‘safety experiences’ may become a quality aspect for travelers’ decision making in the future, even with the treatment availability for COVID-19 or an effective vaccine.

These measures comprise the reinforcement in the use of masks for tourism workers and travelers, an increase in the frequency of cleaning and sanitizing of the establishments, the use of high efficiency particulate arrestance (HEPA) filters to clean the air at places of mass gathering and hotel rooms, the free distribution of personal hygiene kits, automatic hand sanitizer dispensers distributed in areas of high circulation and surface contact, body temperature measurement as a protocol to enter tourism establishments and even borders, and sanitary mats for shoe hygiene.

We believe that other preventive measures that induce a sense of security for travelers will be the new protocol for the reopening of travels and may also contribute to the decrease in the incidence of other viral respiratory infections.

However, we must avoid the use of technologies or substances that, despite being disinfectants, can cause damage or hurt the traveler, such as the use of UV lamps, ozone, and other sanitizers.

The surveillance of infectious diseases and safety experience must be planned by nations and tourism establishments based on the scientific evidence in order to ensure safety of travelers. This must become a major public health policy to avoid infectious disease transmission in future pandemic situations.

## AUTHOR CONTRIBUTIONS

All of the authors affirm that they have contributed to the development of the manuscript. Nali LHS designed the study, collected data and discussed the results. Salvador FS contributed to the investigation, methodology, and manuscript review. Bonani GSS contributed to the investigation and discussion. Andrade Júnior HF contributed to the study design and manuscript review. Luna EJA performed the analysis and manuscript review. Fujita DM designed the study, collected data, and wrote and edited the manuscript.
